# Decoy receptor 3 as a prognostic biomarker for sepsis and septic shock according to the Sepsis-3 definitions

**DOI:** 10.3389/fcimb.2025.1529917

**Published:** 2025-03-07

**Authors:** Long Chen, Xiao Lin, Xing Yu, Chunxia Yang, Rui Li, Qingqing Guo, Jingshi Shi, Xiuyu Liao, Xiaoli Chen, Zengyi Ma, Jiandong Lin

**Affiliations:** ^1^ Department of Neurosurgery & Neurocritical Care, Huashan Hospital, Fudan University, Shanghai, China; ^2^ Intensive Care Unit, the First Affiliated Hospital, Fujian Medical University, Fuzhou, China; ^3^ Department of Gastroenterology, the First Affiliated Hospital, Fujian Medical University, Fuzhou, China

**Keywords:** biomarker, decoy receptor 3, sepsis, septic shock, organ dysfunction, prognosis

## Abstract

**Objectives:**

The present study was conducted to reappraise the prognostic value of decoy receptor 3 (DcR3) for patients with sepsis and septic shock according to the latest Sepsis-3 definitions.

**Methods:**

Subjects suffering from sepsis or septic shock were enrolled within 6 h of admission. The Sequential Organ Failure Assessment (SOFA) score and the plasma levels of DcR3, C-reactive protein, procalcitonin, and interleukin-6 were measured. Group comparisons were made based on the survival status on day 28 after onset. Predictors of mortality were assessed using the Cox proportional hazard models, and survival curves were plotted with the Kaplan–Meier method. Discriminative performances of single and combined indicators were evaluated via the areas under receiver operating characteristic curves.

**Results:**

Among 143 eligible sepsis cases, 77 developed septic shock, and the 28-day mortality rates were 32.2% and 45.5%, respectively. Regardless of the population (all sepsis or septic shock), non-survivors exhibited significantly higher DcR3 levels compared to survivors (median 4.19 vs. 2.64 ng/mL and 4.37 vs. 3.18 ng/mL, respectively; *p* < 0.001 and p = 0.002, respectively). DcR3 levels were most correlated with organ dysfunction presented by SOFA scores (correlation coefficient = 0.347 and 0.308, respectively; p = 0.001 and 0.016, respectively) but did not differ among the various pathogenic microbes of infection. Multivariate Cox regression identified DcR3 as an independent predictor of mortality [hazard ratio (95% confidence interval): 1.570 (1.048–2.352) and 1.828 (1.047–3.194), respectively; p = 0.029 and 0.034, respectively]. Kaplan–Meier analysis showed that elevated DcR3 concentrations were associated with significantly lower survival rates (p = 0.001 and 0.013, respectively). The areas under receiver operating characteristic curves of DcR3 alone for predicting outcome were superior to that of the other three biomarkers (0.731 and 0.711, respectively) and could be further improved when coupled with SOFA scores (0.803 and 0.784, respectively).

**Conclusions:**

DcR3 is a valuable prognostic biomarker for sepsis and septic shock, offering the potential to predict 28-day mortality in clinical settings.

## Introduction

Worldwide, sepsis has been regarded as one of the most life-threatening states and a major public health issue (Meyer and Prescott, 2024). Its updated definition, “organ dysfunction caused by a dysregulated host response to infection” (Sepsis-3), reflects an improved understanding of pathophysiology (Singer et al., 2016). Nevertheless, the clinical practices of sepsis and septic shock still face formidable challenges (de Backer et al., 2024), partly because the derived criteria based on the Sequential Organ Failure Assessment (SOFA) score may come at the expense of sensitivity, leading to delayed discrimination and intervention (Rhee and Klompas, 2017; Lambden et al., 2019). Early prognostic prediction is essential to assign risk profiles and establish treatment intensity, thereby modifying the outcome of patients (Povoa et al., 2023; Cajander et al., 2024).

Over the last decade, biomarkers have been implemented in sepsis to aid decision-making. A biomarker is a characteristic by which a specific physiological or pathological process can be recognized (Biomarkers Definitions Working G, 2001). Several analytes, including C-reactive protein (CRP), procalcitonin (PCT), and interleukin (IL)-6, turn into common markers of acute inflammatory response (Faix, 2013; Tziolos et al., 2015). Furthermore, novel candidates with different biological signatures are explored to help improve the effectiveness of existing assays (Pierrakos et al., 2020).

Decoy receptor 3 (DcR3), also named tumor necrosis factor (TNF) receptor superfamily member 6b, is a soluble receptor for three corresponding ligands involved in apoptosis and inflammation: Fas ligand (FasL), herpes virus entry mediator-L (LIGHT), and TNF-like molecule 1A (TL1A) (Pitti et al., 1998; Yu et al., 1999; Migone et al., 2002). In addition to its neutralizing ability, it directly regulates the differentiation and maturation of myeloid cells, biases the phenotype of helper T cells, inhibits the proliferation of B cells, and mediates the interaction of immune cells (Hsieh and Lin, 2017). The pleiotropic immunomodulator could be exclusively released by antigen-presenting cells (APCs), such as monocytes and dendritic cells (DCs), via the Toll-like receptor (TLR) 2/4-mediated mitogen-activated protein kinase (MAPK) pathway in response to bacterial antigens (Kim et al., 2004). A similar phenomenon was observed in treated human intestinal epithelial cells (IECs) as well (Kim et al., 2005). Increased DcR3 levels have been associated with the severity and outcome of acute respiratory distress syndrome (Chen et al., 2009), coronary artery disease (Chang et al., 2015), kidney diseases (Hung et al., 2012; Maruyama et al., 2016), and liver failure (Lin et al., 2019). In severe burns, non-survivors exhibited a fluctuating pattern of persistently high plasma DcR3 within 31-day monitoring (Min et al., 2021).

The potential of DcR3 as a biomarker for sepsis was also estimated. We previously reported a remarkable elevation of sera DcR3 that could distinguish patients meeting the Sepsis-2 criteria from non-infectious systemic inflammatory response syndrome (SIRS) and correlate with the Acute Physiology and Chronic Health Evaluation (APACHE) II score and PCT (Hou et al., 2012; Kim et al., 2012; Gao et al., 2018). Zhao et al (Zhao et al., 2018) suggested that when combined with PCT and soluble urokinase-type plasminogen activator receptor (suPAR), DcR3 enhanced the efficiency of sepsis diagnosis. Nevertheless, the clinical significance of biomarkers should be reinterpreted as the dedication in identifying sepsis has been shifted from an existing infection to a dysregulated inflammation. In this scenario, we conduct the present study to reappraise the prognostic value of DcR3 in patients suffering from sepsis and septic shock with the Sepsis-3 definitions.

## Methods

### Patients

This prospective cohort study was conducted in the intensive care unit (ICU) of the First Affiliated Hospital of Fujian Medical University from September 2016 to May 2019, with the approval of the University Institutional Ethics Committee (Approval Number: FMU2016142). Adult (≥18 years of age) patients who developed sepsis within 6 h were potentially eligible. The exclusion criteria were as follows: being pregnant and having malignant tumors, severe immunodeficiency, and chronic autoimmune and inflammatory diseases. Informed consent was acquired from the patients enrolled or their legally authorized representatives.

### Diagnosis and treatments

The diagnosis and treatments for sepsis and septic shock followed the international consensus and guidelines updated in 2016 (Singer et al., 2016; Rhodes et al., 2017). Briefly, sepsis was confirmed if patients developed an infection associated with an increase in SOFA score of ≥2 points, and septic shock was accordingly described as sepsis cases with refractory hypotension (mean arterial pressure < 65 mmHg) requiring vasopressors and elevated serum lactate concentration (>2 mmol/L) in the absence of hypovolemia. Once identified, fluid resuscitation, antimicrobial therapy, and other standard management procedures were then initiated without any intervention from the investigators.

### Data collection

Patients received an initial clinical assessment at enrolment, including demographics, comorbidities, and drug usage. The burden of comorbidities was assessed using the Charlson comorbidity index (CCI) derived from chart review (Stavem et al., 2017). Vital signs (consciousness, heart rate, respiratory rate, blood pressure, arterial oxygen saturation, etc.) and routine laboratory tests (white blood cells, platelets, bilirubin, serum creatinine, arterial lactate, etc.) were collected within 1 h of admission to calculate SOFA scores. The sources and pathogens of infection were determined by the attending physicians according to clinical features, microbiological cultures, and the patient responses to antibiotic therapy. The outcome was evaluated by the survival status on day 28 after onset. Follow-up was acquired by medical records or telephone interview if one patient was discharged or transferred to other departments within 28 days.

### Biomarker measurements

Residual blood samples obtained during the course of laboratory testing were stored at −80°C until analysis. Four inflammatory biomarkers were measured: DcR3, CRP, PCT, and IL-6. A quantitative enzyme-linked immunosorbent assay (ELISA) for DcR3 was established as previously reported, with a detection limit of 36 pg/mL (Chen et al., 2004). All samples were tested in the same batch to avoid interassay variation. The DcR3 standards were run simultaneously in the same assay for the unknown calculations. The intra-CVs of assays were <5%–10% and the inter-CVs were <8%–15%. PCT and IL-6 were determined using the Cobas E601 analyzer (Roche, Mannheim, Germany), while CRP was measured with the BN II System (Siemens, Marburg, Germany), as per assay manufacturer instructions.

### Statistical analysis

All variables were presented in a proper form [number with percentage, mean with standard deviation (SD), or median with interquartile range (IQR)]. Based on assumption validity, comparisons were made using the t-test or Mann–Whitney U test for continuous data and the chi-square or Fisher’s exact test for categorical data. Pearson or Spearman correlation analysis served to analyze the association between two variables. A Cox proportional hazard regression model was conducted for multivariate analyses of 28-day mortality, and hazard ratios (HRs) and the corresponding 95% confidence intervals (CIs) are presented. The SOFA scores and all four biomarker levels were entered separately into the Cox model, taking into account their potential relevance. Where applicable, adjustments were made for age, sex, and possible confounding clinical variables (CCI and septic shock status). The survival analyses of patients with different DcR3 levels were performed using the Kaplan–Meier method and the log-rank test. Receiver operating characteristic (ROC) curves were computed and areas under the curves (AUCs) were calculated to assess the predictive value of indicators to patient outcomes. Bootstrapping with multiple iterative resamples was applied to compute the variability and generate CIs for ROC curves (Henderson, 2005). A combined prediction was achieved by constructing a multivariate logistic regression model, which consisted of a constant term and selected variables assigned with respective weight coefficients.

All calculations were accomplished with R programming (R Foundation for Statistical Computing) and GraphPad Prism 8.0 (GraphPad Software, La Jolla, CA, USA). Statistical significance was set at the level of p-value <0.05.

## Results

### Characteristics of the patients

**Figure 1:**
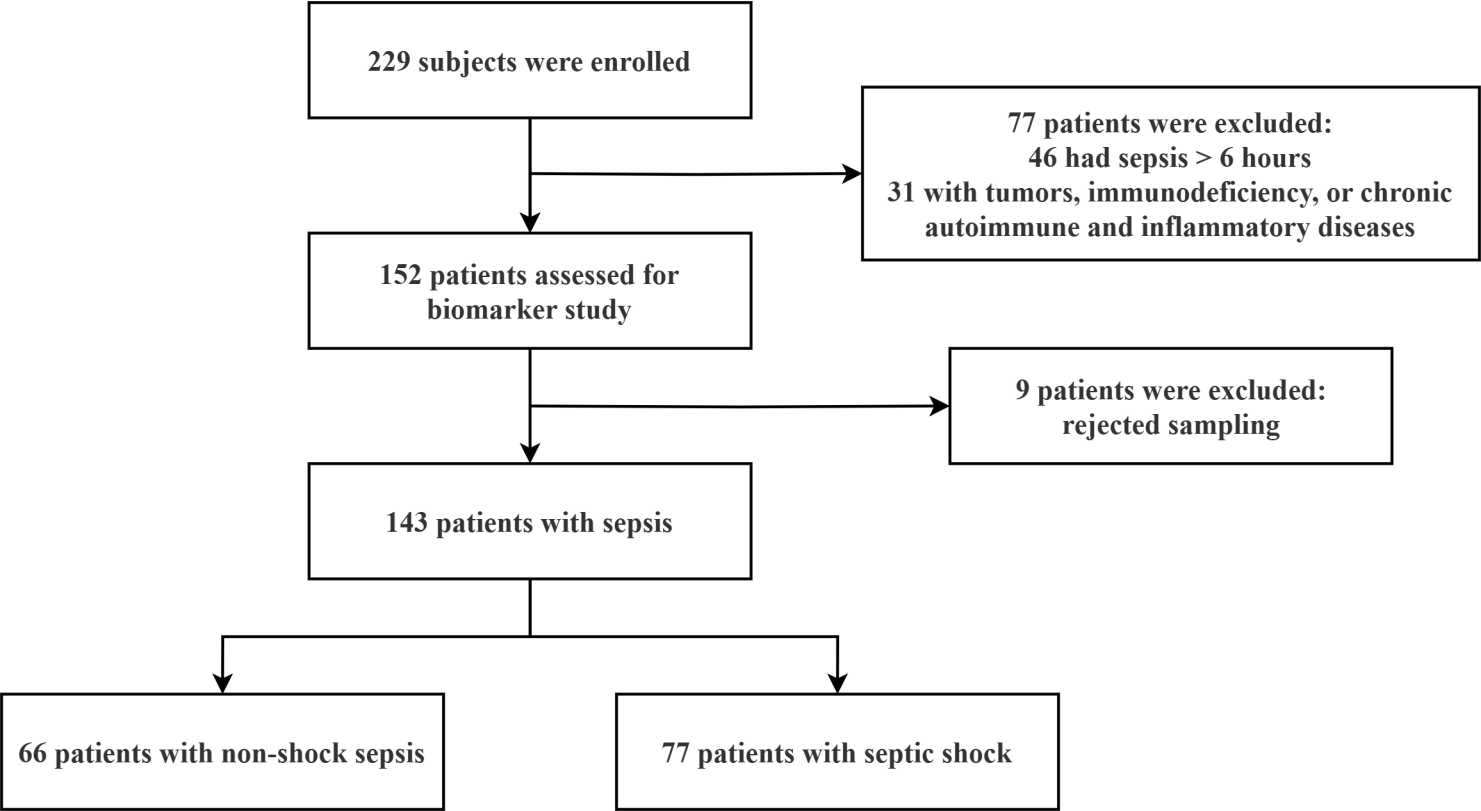
Flowchart of the study participants.

A total of 143 patients with sepsis were enrolled in the final analysis, of which 77 cases met the diagnostic criteria of septic shock (**Figure 1**). The demographics, clinical characteristics, and laboratory results of the subjects are summarized in **Table 1**. On day 28, the mortality was 32.2% (46/143) for all sepsis and 45.5% (35/77) for the subset of septic shock. Non-survivors underwent a considerably shortened median (IQR) survival time [7 (3, 10) days in both populations, *p* < 0.001] and had an older mean (SD) age [70.0 (15.0) vs. 61.7 (18.2) for all sepsis, p = 0.005; 70.9 (15.5) vs. 61.6 (16.3) for septic shock, p = 0.013], more commonly documented heart failure (58.7% vs. 32.0% for all sepsis, p = 0.002; 57.1% vs. 26.2% for septic shock, p = 0.006), and higher median (IQR) Charlson comorbidity index [2 (1, 4) vs. 1 (0, 3) for all sepsis, p = 0.006; 2 (1, 4) vs. 1 (1, 2) for septic shock, p = 0.026] and SOFA score [11 (6, 16) vs. 6 (4, 8) for all sepsis, *p* < 0.001; 11 (6, 16) vs. 7 (5, 10) for septic shock, p = 0.002]. For the entire cohort, non-survivors tended to present a state of septic shock initially compared to survivors (76.1% vs. 43.3%, *p* < 0.001) and were more often accompanied by chronic kidney disease (34.8% vs. 13.4%, p = 0.003). Among patients with septic shock, infections originating from the abdomen were more lethal (45.7% vs. 23.8%, p = 0.043).

**Table 1:** Demographics, clinical characteristics, laboratory findings, and outcomes of the subjects.

	All sepsis			Septic shock		
	Survivor (*n* = 97)	Non-survivor (*n* = 46)	*p*-value	Survivor (*n* = 42)	Non-survivor (*n* = 35)	*p*-value
Age, years, mean ± SD	61.7 ± 18.2	70.0 ± 15.0	0.005	61.6 ± 16.3	70.9 ± 15.5	0.013
Male, *n* (%)	66 (68.0)	33 (71.7)	0.654	28 (66.7)	25 (71.4)	0.653
Comorbidity, *n* (%)						
Chronic obstructive pulmonary disease	18 (18.6)	4 (8.7)	0.127	6 (14.3)	3 (8.6)	0.437
Congestive heart failure	31 (32.0)	27 (58.7)	0.002	11 (26.2)	20 (57.1)	0.006
Stroke	28 (28.9)	16 (34.8)	0.474	12 (28.6)	13 (37.1)	0.424
Hepatobiliary and pancreatic diseases	11 (11.3)	8 (17.4)	0.319	5 (11.9)	8 (22.9)	0.201
Gastrointestinal diseases	6 (6.2)	4 (8.7)	0.582	4 (9.5)	3 (8.6)	0.885
Chronic kidney disease	13 (13.4)	16 (34.8)	0.003	6 (14.3)	11 (31.4)	0.071
Diabetes	26 (26.8)	13 (28.3)	0.855	9 (21.4)	9 (25.7)	0.658
Trauma	10 (10.3)	3 (6.5)	0.462	4 (9.5)	2 (5.7)	0.535
Charlson comorbidity index, median (IQR)	1 (0, 3)	2 (1, 4)	0.006	1 (1, 2)	2 (1, 4)	0.026
Septic shock, *n* (%)	42 (43.3)	35 (76.1)	<0.001	–	–	–
Infection site, *n* (%)						
Lung	30 (30.9)	16 (34.8)	0.645	14 (33.3)	11 (31.4)	0.859
Central nervous system	7 (7.2)	2 (4.3)	0.509	2 (4.8)	1 (2.9)	0.667
Abdomen	25 (25.8)	18 (39.1)	0.104	10 (23.8)	16 (45.7)	0.043
Genitourinary tract	20 (20.6)	6 (13.0)	0.273	11 (26.2)	4 (11.4)	0.103
Skin, skeleton, and soft issue	13 (13.4)	8 (17.4)	0.529	7 (16.7)	5 (14.3)	0.774
Unknown	10 (10.3)	8 (17.4)	0.233	5 (11.9)	6 (17.1)	0.513
Positive blood cultures, *n* (%)	54 (55.7)	25 (54.3)	0.882	27 (64.3)	18 (51.4)	0.254
Pathogens, *n* (%)						
Gram-positive bacteria	29 (29.9)	14 (30.4)	0.948	11 (26.2)	8 (22.9)	0.735
Gram-negative bacteria	48 (49.5)	25 (54.3)	0.587	21 (50.0)	20 (57.1)	0.532
Fungi	18 (18.6)	7 (15.2)	0.623	9 (21.4)	4 (11.4)	0.243
Viruses	1 (1.0)	0 (0.0)	0.490	1 (2.4)	0 (0.0)	0.358
Polymicrobial infection, *n* (%)	15 (15.5)	9 (19.6)	0.540	6 (14.3)	6 (17.1)	0.731
Inconclusive microbiological evidence, *n* (%)	16 (16.5)	11 (23.9)	0.290	6 (14.3)	10 (28.6)	0.124
Laboratory findings, mean ± SD or median (IQR)						
White blood cells, 10^9^/L	12.8 ± 5.6	14.2 ± 8.7	0.320	13.3 ± 5.7	14.7 ± 9.2	0.446
Platelets, 10^9^/L	156.0 (89.0, 221.5)	120.0 (65.8, 202.8)	0.256	133.5 (65.5, 196.5)	112.0 (66.0, 196.0)	0.771
Bilirubin, mg/dL	10.3 (6.7, 19.4)	14.1 (8.3, 30.3)	0.051	11.4 (6.4, 21.3)	14.4 (8.8, 35.7)	0.076
Creatinine, mg/dL	86.6 (53.1, 138.9)	125.2 (79.4, 204.8)	0.004	95.2 (62.7, 159.3)	109.0 (77.1, 182.5)	0.218
Lactate, mmol/L	2.64 (1.98, 3.58)	4.21 (2.95, 6.94)	<0.001	3.46 (2.62, 4.49)	4.34 (3.12, 8.38)	0.066
SOFA score, median (IQR)	6 (4, 8)	11 (6, 16)	<0.001	7 (5, 10)	11 (6, 16)	0.002
Follow-up period, days, median (IQR)	28 (28, 28)	7 (3, 10)	<0.001	28 (28, 28)	7 (3, 10)	<0.001

SD, standard deviation; IQR, interquartile range; SOFA, Sequential Organ Failure Assessment.

### Plasma biomarker levels in patients with sepsis and septic shock

**Table 2** lists the median (IQR) levels of plasma DcR3, CRP, PCT, and IL-6. Sepsis non-survivors displayed generally higher concentrations of all four proteins. However, only DcR3 levels were consistently different between non-survivors and survivors of septic shock with statistical significance [4.37 (3.04, 6.93) vs. 3.18 (2.16, 4.33) ng/mL, p = 0.002], while the rest of the indicators were overlapping and failed to distinguish the two groups.

**Table 2:** Biomarker measurements in patients with all sepsis and septic shock subgroups.

Biomarkers, median (IQR)	All sepsis			Septic shock		
	Survivor (*n* = 97)	Non-survivor (*n* = 46)	*p*-value	Survivor (*n* = 42)	Non-survivor (*n* = 35)	*p*-value
DcR3, ng/mL	2.64 (2.00, 3.65)	4.19 (2.82, 5.73)	<0.001	3.18 (2.16, 4.33)	4.37 (3.04, 6.93)	0.002
C-reactive protein, mg/L	79.20 (52.56, 90.00)	90.00 (65.70, 113.75)	0.025	88.41 (63.57, 115.75)	90.00 (68.78, 125.00)	0.430
Procalcitonin, ng/mL	2.97 (0.71, 20.08)	5.88 (3.06, 40.43)	0.002	7.11 (0.78, 45.18)	7.86 (3.96, 58.50)	0.102
Interleukin-6, pg/mL	198.10 (59.50, 1,601.00)	877.55 (145.35, 3,544.75)	0.008	737.85 (175.15, 3,611.50)	1,556.00 (211.40, 4,591.00)	0.242

IQR, interquartile range.

Spearman’s rank test was further performed to correlate the biomarker levels with organ dysfunction presented by SOFA scores (**Table 3**). DcR3 showcased the most pronounced association with the SOFA score (correlation coefficient = 0.347, p = 0.001) among patients with sepsis. Additionally, a significant concordance was exclusively observed between DcR3 and the SOFA score in individuals experiencing septic shock (correlation coefficient = 0.308, p = 0.016).

**Table 3:** Correlations between biomarker levels and SOFA score in all sepsis and septic shock subgroups.

Variable	All sepsis		Septic shock	
	Correlation coefficient	*p*-value	Correlation coefficient	*p*-value
Decoy receptor 3	0.347	0.001	0.308	0.016
C-reactive protein	0.145	0.083	0.063	0.585
Procalcitonin	0.199	0.017	0.221	0.053
Interleukin-6	0.264	0.001	0.087	0.453

SOFA, Sequential Organ Failure Assessment.

**Figure 2:**
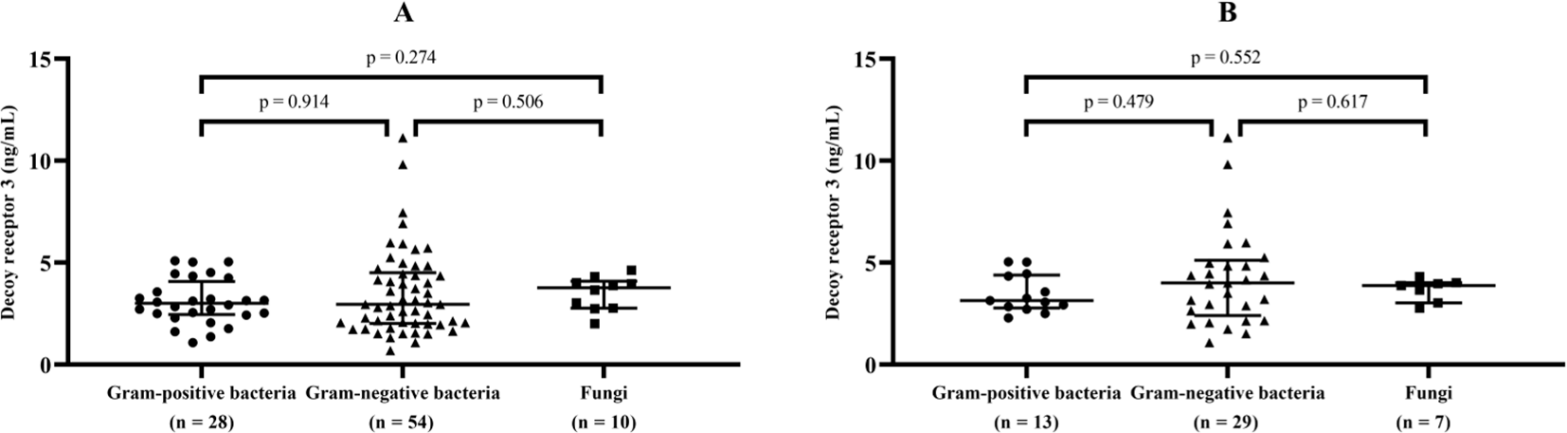
DcR3 levels in sepsis and septic shock caused by different types of pathogens. Median levels and interquartile range of decoy receptor 3 (marked by transverse lines) were compared in sepsis **(A)** and septic shock **(B)** with isolated Gram-positive, Gram-negative, or fungal infections.

To investigate the host response to different types of pathogenic microorganisms, DcR3 levels were compared after ruling out 24 cases of multiple infections (including the only case of viral and bacterial co-infection) and 27 cases without conclusive microbiological evidence. For sepsis caused by isolated Gram-positive, Gram-negative, or fungal infections (92 cases), the median (IQR) levels of DcR3 were 3.00 (2.45, 4.09), 2.96 (2.02, 4.51), and 3.77 (2.76, 4.10) ng/mL, respectively; in patients who developed septic shock (49 cases), the corresponding median (IQR) levels of DcR3 were 3.15 (2.78, 4.40), 4.00 (2.40, 5.12), and 3.88 (3.03, 4.02) ng/mL, respectively. No significant differences were found among the above subgroups (**Figure 2**).

### DcR3 as an independent risk factor for mortality in sepsis and septic shock

The results of the multivariate Cox regression analysis are shown in **Table 4**. To reduce the right skewness, the SOFA scores and biomarker levels were natural log-transformed and further standardized before inclusion as continuous variables. For the overall subjects, the SOFA score and DcR3 and PCT levels were independently associated with the 28-day mortality [HR (95% CI) 1.833 (1.257–2.674), 1.570 (1.048–2.352), and 1.374 (1.011–1.869); p = 0.002, 0.029, and 0.043, respectively], after adjusting for age, sex, CCI, and septic shock status. In the septic shock subgroup, DcR3 achieved an HR (95% CI) of 1.828 (1.047–3.194), as the only significant risk factor for 28-day mortality among the four biomarkers (p = 0.034), with adjustment for age, sex, and CCI.

**Table 4:** Cox proportional hazard models for predicting 28-day mortality in all sepsis and septic shock subgroups.

Variable	All sepsis^a^		Septic shock^b^	
	HR (95% CI)	*p*-value	HR (95% CI)	*p*-value
SOFA score	1.833 (1.257, 2.674)	0.002	1.651 (1.065, 2.559)	0.025
Decoy receptor 3	1.570 (1.048, 2.352)	0.029	1.828 (1.047, 3.194)	0.034
C-reactive protein	1.181 (0.846, 1.649)	0.328	1.106 (0.711, 1.719)	0.656
Procalcitonin	1.374 (1.011, 1.869)	0.043	1.386 (0.984, 1.953)	0.062
Interleukin-6	1.153 (0.811, 1.639)	0.428	1.167 (0.768, 1.773)	0.471

All variables were natural log-transformed and further standardized (i.e., per standard deviation increase) in Cox regression models.

HR, hazard ratio; CI, confidence interval; SOFA, Sequential Organ Failure Assessment.

a Adjusted for age (in continuous), gender, Charlson comorbidity index, and septic shock.

b Adjusted for age (in continuous), gender, and Charlson comorbidity index.

**Figure 3:**
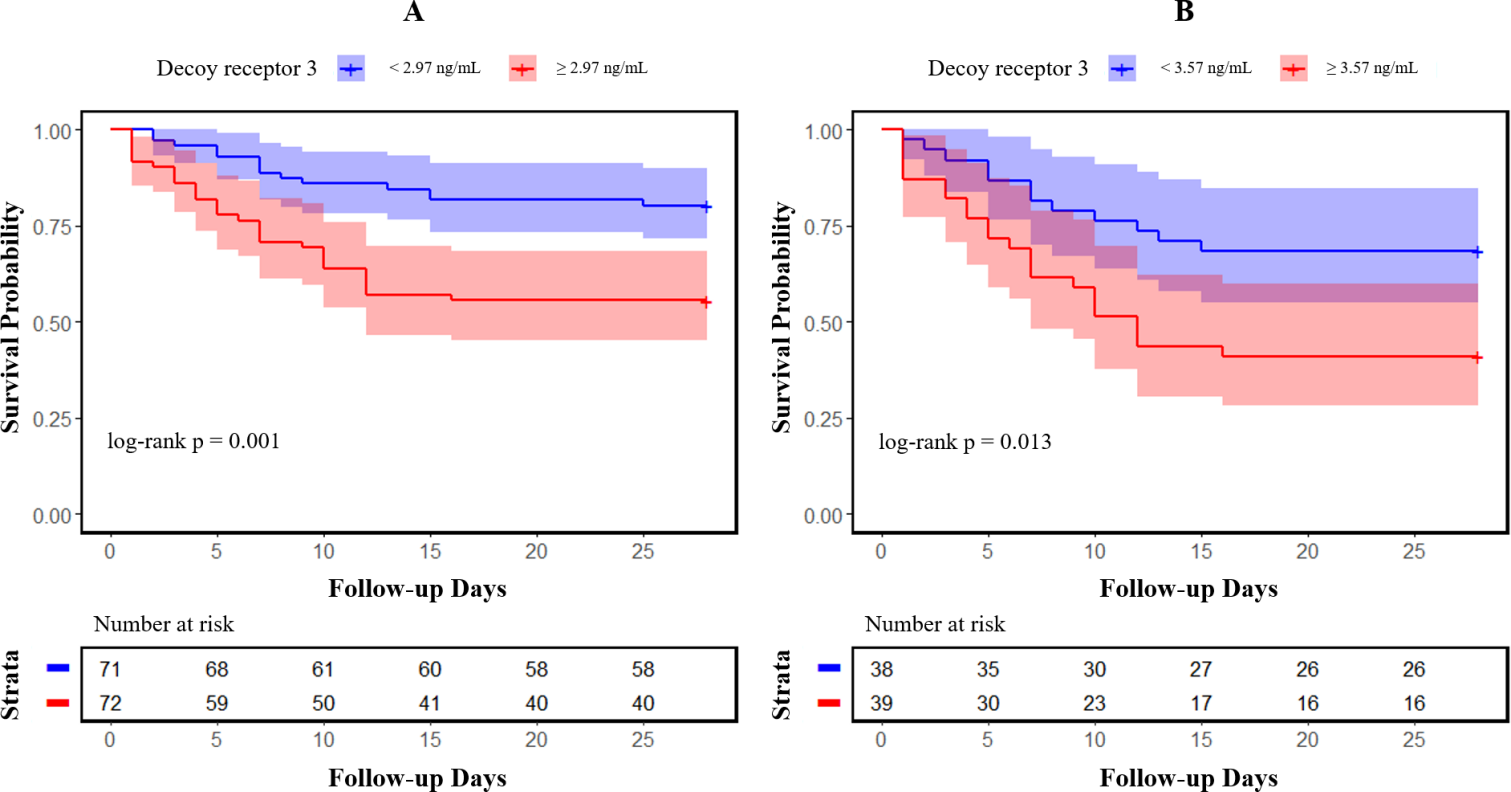
Kaplan–Meier survival curves of 28-day mortality in patients with sepsis and septic shock. Patients were categorized into two groups based on the median value of decoy receptor 3 levels in sepsis **(A)** and septic shock **(B)**; 95% confidence intervals are presented as semitransparent zones.

Categorized by the median value of DcR3 levels (2.97 ng/mL for sepsis and 3.57 ng/mL for septic shock, respectively), Kaplan–Meier survival curves with log-rank tests clearly illustrated that poorer patient outcomes were associated with higher DcR3 expression in sepsis (**Figure 3A**, p = 0.001) and septic shock (**Figure 3B**, p = 0.013).

### Outcome prediction by DcR3 in patients with sepsis and septic shock

**Figure 4:**
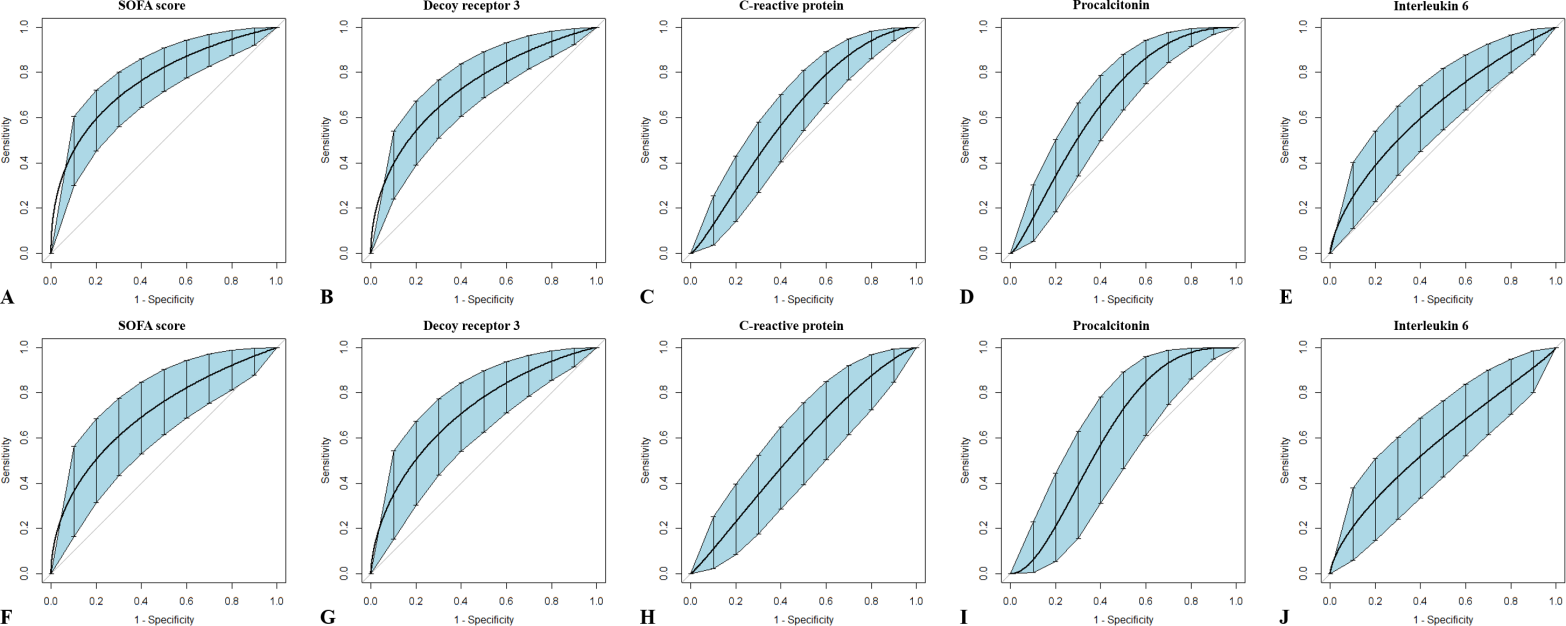
Receiver operating characteristic curves of single indicators for outcome prediction in patients with sepsis or septic shock. Sepsis **(A–E)** and septic shock **(F–J)** are variously discriminated by Sequential Organ Failure Assessment scores, and plasma levels of decoy receptor 3, C-reactive protein, procalcitonin, and interleukin-6 were measured within 6 h of onset. Bootstrapping with 1,000 resamples was utilized to smooth the curves and generate 95% confidence intervals.

The ROC curves for predicting mortality using the SOFA scores and plasma levels of the four markers are plotted in **Figure 4**, and the corresponding AUCs and optimal cutoff values are listed in **Table 5**. In view of the insufficient number of subjects, bootstrapping with 1,000 resamples was performed to provide a robust estimate of the uncertainty in the curves. To the sepsis subgroup, the AUCs (95% CIs) in descending order were 0.749 (0.669–0.817) for SOFA score, 0.731 (0.650–0.801) for DcR3, 0.658 (0.574–0.736) for PCT, 0.637 (0.552–0.715) for IL-6, and 0.617 (0.532–0.697) for CRP; to the septic shock subgroup, the AUCs (95% CIs) were 0.711 (0.596–0.808) for DcR3, 0.704 (0.590–0.803) for SOFA score, 0.609 (0.491–0.718) for PCT, 0.578 (0.460–0.689) for IL-6m and 0.552 (0.435–0.666) for CRP. Although all parameters were able to predict the 28-day mortality in sepsis, only SOFA score and DcR3 remained competent in septic shock (both p = 0.002).

**Table 5:** AUCs for indicators to discriminate 28-day mortality in all sepsis and septic shock subgroups.

Variable	AUC (95% CI)	*p*-value	Optimal cutoff value	Sensitivity (%)	Specificity (%)
All sepsis, *n* = 143					
SOFA score	0.749 (0.669–0.817)	<0.001	9	56.52	85.57
Decoy receptor 3	0.731 (0.650–0.801)	<0.001	3.89	56.52	81.44
C-reactive protein	0.617 (0.532–0.697)	0.025	75.83	71.74	49.48
Procalcitonin	0.658 (0.574–0.736)	0.002	1.02	95.65	35.05
Interleukin-6	0.637 (0.552–0.715)	0.008	517.80	60.87	64.95
Septic shock, *n* = 77					
SOFA score	0.704 (0.590–0.803)	0.002	13	42.86	92.86
Decoy receptor 3	0.711 (0.596–0.808)	0.002	3.88	65.71	69.05
C-reactive protein	0.552 (0.435–0.666)	0.431	55.23	100.00	19.05
Procalcitonin	0.609 (0.491–0.718)	0.102	2.00	97.14	38.10
Interleukin-6	0.578 (0.460–0.689)	0.244	2,698.00	42.86	73.81

AUC, area under the curve; CI, confidence interval; SOFA, Sequential Organ Failure Assessment.

**Figure 5:**
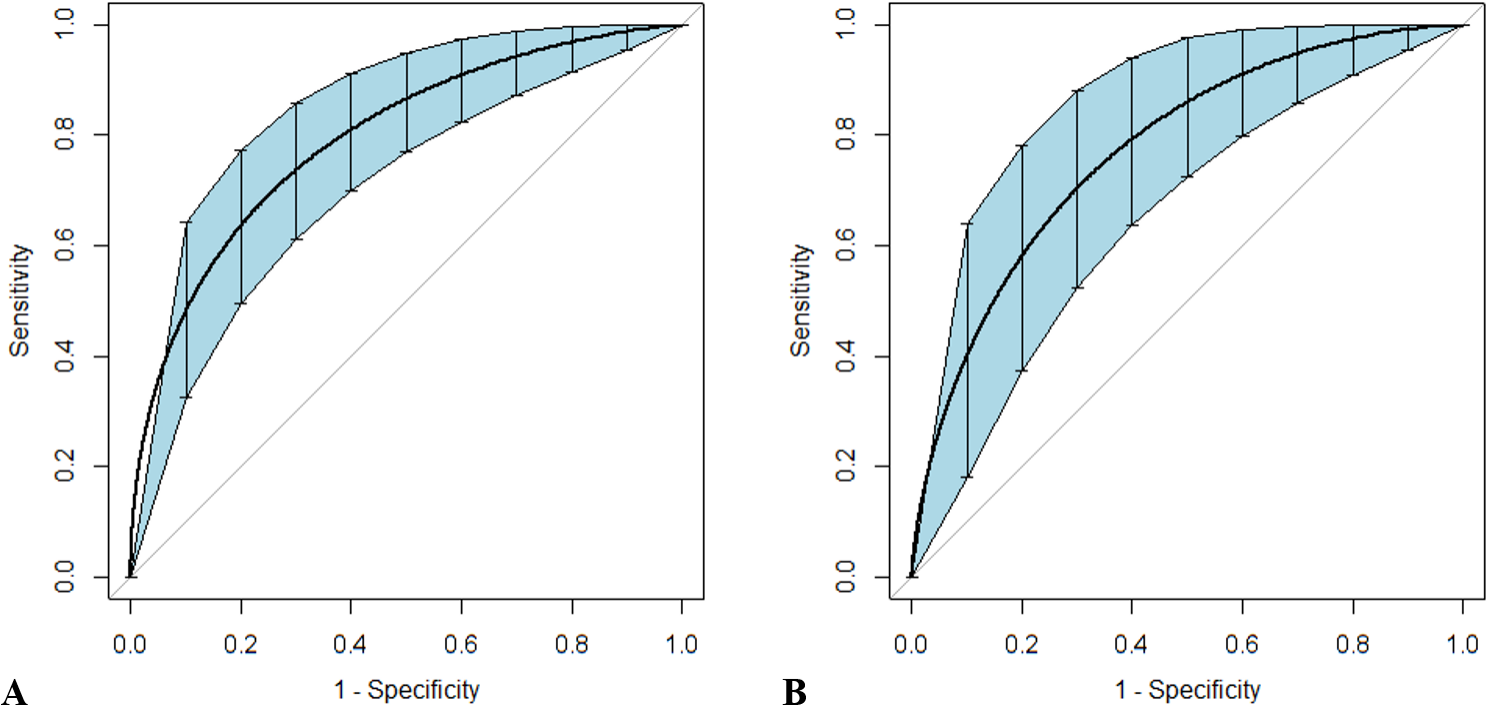
Receiver operating characteristic curves for the combined probe constructed to predict outcomes in patients with sepsis and septic shock. Sepsis **(A)** and septic shock **(B)** are discriminated by the combination of Sequential Organ Failure Assessment scores and decoy receptor 3. Curves were smoothed by bootstrapping with 1,000 resamples, and 95% confidence intervals are displayed.

Since DcR3 and SOFA score were the only two indicators that showed fair discriminative performance by AUC (over 0.70), the logistic regression equation was established based on their combination. The joint probe was found to remarkably enhance the predictive efficiency of patient outcomes in sepsis [AUC (95% CI), 0.803 (0.728–0.865)] and septic shock [AUC (95% CI), 0.784 (0.676–0.870); **Figure 5**, **Table 6**].

**Table 6:** AUCs for the combined probe to discriminate mortality in all sepsis and septic shock subgroups.

Subjects	AUC (95% CI)	*p*-value	Optimal cutoff value	Sensitivity (%)	Specificity (%)
All sepsis, *n* = 143	0.803 (0.728–0.865)	<0.001	–	78.26	74.23
Septic shock, *n* = 77	0.784 (0.676–0.870)	<0.001	–	71.43	80.95

AUC, area under the curve; CI, confidence interval; SOFA, Sequential Organ Failure Assessment.

## Discussion

The current study has supported DcR3 as an important prognostic factor in the early development of sepsis and septic shock. Proofs include the following: 1) the plasma DcR3 levels exhibited a significant disparity between survivors and non-survivors in patients with sepsis or septic shock; 2) the DcR3 levels were positively correlated with organ dysfunction as measured by the SOFA score; 3) among the four biomarkers, DcR3 was the only one independently associated with the 28-day mortality in both cohorts; and 4) the efficacy of DcR3 alone for outcome prediction was credible, which could be further improved when coupled with SOFA score.

It is another exploration on clinical significance of this molecule in sepsis, which has been allocated a fresh task according to the latest definitions. Rather than seeking conclusive microbiological evidence, practices in sepsis nowadays focus on the timely recognition of patients who may progress to a critical state (Singer et al., 2016). We herein adopted a stepwise and systematic statistical approach to visit the prognostic value of DcR3 and other parameters, providing more detailed information to help personalize management. Notably, the data enrolled in the analysis were mainly measured within the first 6 h of onset, with the belief that assigning risk profiles and predicting outcome as early as possible could be more beneficial (Povoa et al., 2023; Cajander et al., 2024). We previously reported two dynamic trends of DcR3 in individuals who died from sepsis, i.e., a sustained rise or an initial peak followed by a decline. Nevertheless, once the molecule reached a certain threshold, clinical outcomes would be poor regardless of the pattern (Gao et al., 2018).

The dysregulated immune responses and organ dysfunctions highlighted by the revised definition remain difficult to assess. Despite the validity to clinically characterize patients with sepsis, the SOFA score is less sensitive and is a complicated algorithm (Rhee and Klompas, 2017; Lambden et al., 2019). Analysis from the Medical Information Mart for Intensive Care-III (MIMIC-III) database showed that the definitions of Sepsis-3 may have narrowed the sepsis population in comparison with Sepsis-1, leading to false negatives. Among patients who did not satisfy the new diagnostic criteria, the fatality rate was over 6.0% (Fang et al., 2018). In a cohort with severe Gram-negative infections, the earliest time point for change in SOFA score to determine prognosis was the 7th day after sepsis, implying a dullness in the recognition of illness (Karakike et al., 2019). With a well-established ELISA, soluble DcR3 could be efficiently and promptly determined across a variety of human biological fluids, proving its broad clinical applicability (Chen et al., 2004). The rising concentration was not only linked to the occurrence and progression of the acute inflammations previously enumerated but could also be seen in chronic illnesses and tumors (Wu et al., 2003; Connor and Felder, 2008; Aiba et al., 2014; Xiu et al., 2015). In specific contexts, its levels may serve as a basis for distinguishing different pathophysiological states. During the Sepsis-2 era, we have serially reported the ability of a remarkable elevation of serum DcR3 to discriminate sepsis from SIRS, as well as its correlation with the severity of infection (Hou et al., 2012; Kim et al., 2012; Gao et al., 2018). In this study, using the marker alone could achieve a similar AUC value to that of using the scoring system for the prediction of 28-day mortality, raising its benefits in clinical settings. Moreover, DcR3 levels appeared most correlated to SOFA scores, hinting at a potential relationship with the advancement of organ dysfunction, as revealed in other studies (Chen et al., 2009).

Compared with the three other important inflammatory biomarkers (i.e., CRP, PCT, and IL-6), DcR3 showed a stronger capacity on outcome prediction, which is consistent with our recent observations from *in-vitro* experiments (unpublished data). When monocytes were stimulated with varying concentrations of lipopolysaccharide (LPS), the levels of most inflammatory factors demonstrated a significant increase under mild conditions, whereas DcR3 only showed an appropriate response to intense stimulation, suggesting it as a more suitable biomarker for sepsis. A proper comprehension of the unique biological effects of this molecule may help interpret the results. Similar to most acute-phase reactants or proinflammatory cytokines, DcR3 can be triggered by pathogens or insults, thus becoming detectable in circulation. However, unlike them, it tends to attenuate inflammation and facilitate tissue repair by suppressing the release of cytotoxic mediators, preventing apoptosis and inducing differentiation (Liang et al., 2015; Su et al., 2024). From a physiological perspective, the upregulation of DcR3 represents a negative feedback mechanism of the host to maintain a moderate immune response and return to homeostasis. However, the product may not be sufficient to eliminate hyperinflammation in a pathological setting (Min et al., 2021). Therefore, the detected level of DcR3 can be regarded as a sign for the dysregulated degree of response and distinguish protective inflammation from pathological cytokine storm (Fajgenbaum and June, 2020). The aforementioned hypothesis plausibly elucidates the paradox that supplementation of DcR3 may serve as a promising immunotherapeutic agent for numerous inflammatory diseases including sepsis, while its endogenous counterpart functions as a biomarker indicative of organ dysfunction and dismal outcomes (Su et al., 2023). The gap between the intensity of inflammation and the amount of molecules involved in the reaction appears to be a crucial component affecting the fate of the host.

In particular, DcR3 was the only biomarker independently associated with 28-day mortality of patients with septic shock and gave the greatest AUC for predicting death. We speculated that the results may be related to the extensive and severe damage of the endothelium, a key element in septic shock pathophysiology. Endothelial dysfunction caused by the innate immune response to infection would bring about impaired microvascular perfusion, inadequate tissue oxygenation, and abnormal cell metabolism and contribute to a decrease in venous return and hence sepsis-induced hypotension. In addition, endothelial alteration is also implicated in the inflammatory activation and coagulation processes leading to the amplification of septic response and development of organ dysfunction (de Backer et al., 2021; Bakker et al., 2022). It has been proven that DcR3 could be upregulated at both the mRNA and protein levels following treatment with different microbial toxins in human umbilical vein endothelial cells (HUVECs) (Yoo et al., 2012; Hou et al., 2018), and interestingly, its ligand TL1A seems to be exclusively expressed in HUVECs rather than human aortic endothelial cells (HAECs), acting as a potent inhibitor to induce endothelial apoptosis and suppress angiogenesis (Yang et al., 2004). This may partially explain the predominance of microcirculatory injury on the venular side during septic shock, but further supportive studies are needed. Recently, we reported a profound elevation of DcR3 in severe burn shock pathologically characterized by massive endothelial damage and capillary leakage. The increase was pronounced by a co-infected state and was associated with the death of subjects that fulfilled the diagnostic criteria for septic shock (Min et al., 2021). Together with previous studies, our findings show that DcR3 can become an expected predictor for the outcome of septic shock.

It should be acknowledged that DcR3 failed to discriminate among sepsis cases caused by diverse pathogens, and none of the biomarkers in our study presented sufficiently convincing predictive power for prognosis. Due to the multifaceted nature of the host response to infection, it is difficult to determine the contribution of different causative microorganisms to DcR3 induction despite different mechanisms (Kim et al., 2004). Likewise, in light of the complicated pathobiology, no single indicator hitherto has been reliable enough for diagnosing sepsis or predicting outcomes. The integration of laboratory parameters with clinical data can provide more specific pathophysiological information, which is promising to overcome the obstacles (Yang et al., 2016; Mearelli et al., 2018). Our results indicate that paired with SOFA score, DcR3 has a good synergistic effect in predicting the prognosis of sepsis and can be used as a good complementary tool with application prospects. Yet, it was not combined with other biomarkers because of their weak predictive capabilities and possible intrinsic relationship with DcR3. The combination of multiple markers has not constantly outperformed an individual one (Parlato et al., 2018).

Patients with coexisting tumors were excluded from our study, given the controversy over whether the presence of tumors affects the level of DcR3 responses in an inflammatory state (Thompson and Connor, 2019). Other underlying diseases were also adjusted in multivariate regression analyses to minimize their impact on outcomes.

### Limitations

The current study may suffer from typical design issues of any single-center cohort, such as a relatively small sample size and the absence of external validation. One possible consequence of the former was that, being the only biomarker with an AUC exceeding 0.70, although DcR3 displayed superior predictive performance over other competitors according to traditional evaluation criteria, this advantage lacked statistical significance (DeLong test). Furthermore, the high mortality, especially in the early stages of the disease, restricted our dynamic tracking of the protein. Finally, no simplification using set cutoffs was recommended since the aim was to reveal the potential value of the novel indicator, and clinical algorithms need to be developed in future research.

## Conclusions

The Sepsis-3 definition requires more precise outcome prediction and risk stratification. The current study discloses the potential of DcR3 as a prognostic biomarker in the early development of sepsis and septic shock by predicting 28-day mortality. Nevertheless, further validation studies are warranted before it can be routinely utilized in the clinical assessment of critically ill patients.

## Data Availability

The datasets presented in this article are not readily available because data will be made available only upon justified request and appropriate approval via formal application to the corresponding author. Requests to access the datasets should be directed to Long Chen (leon_chen@fudan.edu.cn).
